# Automatic User Preferences Selection of Smart Hearing Aid Using BioAid

**DOI:** 10.3390/s22208031

**Published:** 2022-10-20

**Authors:** Hafeez Ur Rehman Siddiqui, Adil Ali Saleem, Muhammad Amjad Raza, Kainat Zafar, Riccardo Russo, Sandra Dudley

**Affiliations:** 1 Institute of Computer Science, Khawaja Fareed University of Engineering and Information Technology, Rahim Yar Khan 64200, Pakistan; 2 Department of Brain and Behavioral Sciences, University of Pavia, 27100 Pavia, Italy; 3 School of Engineering, London South Bank University, London SE1 0AA, UK

**Keywords:** hearing aid, DCASE, machine learning, BioAid, signal processing

## Abstract

Noisy environments, changes and variations in the volume of speech, and non-face-to-face conversations impair the user experience with hearing aids. Generally, a hearing aid amplifies sounds so that a hearing-impaired person can listen, converse, and actively engage in daily activities. Presently, there are some sophisticated hearing aid algorithms available that operate on numerous frequency bands to not only amplify but also provide tuning and noise filtering to minimize background distractions. One of those is the BioAid assistive hearing system, which is an open-source, freely available downloadable app with twenty-four tuning settings. Critically, with this device, a person suffering with hearing loss must manually alter the settings/tuning of their hearing device when their surroundings and scene changes in order to attain a comfortable level of hearing. However, this manual switching among multiple tuning settings is inconvenient and cumbersome since the user is forced to switch to the state that best matches the scene every time the auditory environment changes. The goal of this study is to eliminate this manual switching and automate the BioAid with a scene classification algorithm so that the system automatically identifies the user-selected preferences based on adequate training. The aim of acoustic scene classification is to recognize the audio signature of one of the predefined scene classes that best represent the environment in which it was recorded. BioAid, an open-source biological inspired hearing aid algorithm, is used after conversion to Python. The proposed method consists of two main parts: classification of auditory scenes and selection of hearing aid tuning settings based on user experiences. The DCASE2017 dataset is utilized for scene classification. Among the many classifiers that were trained and tested, random forests have the highest accuracy of 99.7%. In the second part, clean speech audios from the LJ speech dataset are combined with scenes, and the user is asked to listen to the resulting audios and adjust the presets and subsets. A CSV file stores the selection of presets and subsets at which the user can hear clearly against the scenes. Various classifiers are trained on the dataset of user preferences. After training, clean speech audio was convolved with the scene and fed as input to the scene classifier that predicts the scene. The predicted scene was then fed as input to the preset classifier that predicts the user’s choice for preset and subset. The BioAid is automatically tuned to the predicted selection. The accuracy of random forest in the prediction of presets and subsets was 100%. This proposed approach has great potential to eliminate the tedious manual switching of hearing assistive device parameters by allowing hearing-impaired individuals to actively participate in daily life by automatically adjusting hearing aid settings based on the acoustic scene.

## 1. Introduction

Hearing loss (HL) can develop from peripheral or central auditory system damage [1]. Age-related degeneration, genetic mutations, noise exposure, therapeutic drugs with toxic side effects, and chronic disorders induce sensorineural hearing loss [1]. Hearing obstruction means not hearing regular sound frequencies. HL affects one or both ears and can be complete or partial. HL causes psychological and social isolation and also affects how people interact with family and friends, making it harder to perform tasks or study [2]. Around 466 million individuals worldwide suffer from hearing impairment, with 34 million of them being children [3]. According to [4], there are four stages of HL mild, moderate, severe, and profound with the lowest sound perception from 25 to 40 dB, 40 to 70 dB, 71 to 90 dB, and above 91 dB, respectively.

Hearing aids (HAs) and hearing assistive devices (HADs) are devices designed to help hearing-impaired people [5]. HAs and HADs are used to alleviate the symptoms of mild to moderate hearing loss by amplifying sound. The “Array Process digital HA” was an early attempt to develop a digital HA and offers a simulation for investigators to assist in designing Digital HAs [6]. The first effort to customize an HA by [7] used a modified simplex technique, whereas [8] used genetic algorithms to choose settings depending on user reactions.

Noise reduction, beam-forming, feedback cancellation, and speech enhancement are all functions of digital HAs with signal processing [9]. The most difficult challenge in HA is addressing the issue of speech interpretation in acoustic environments. Over the last three decades, there has been a major concentration on the topic of customizing through adjusting system parameters [10].

Smartphones are great for implementing complex signal processing methods, such as hearing testing and refining HA applications. Smartphones are utilized to design and test signal processing algorithms for audiometers and HAs because its user-friendly and growing. Pure-tone audiometry (PTA) and EarTrumpet are smartphone-based Has used by audiologists to remotely adjust patients’ Has [11].

This study’s concept is based on the computer model given in [12] and developed by [13] for the IOS system under the name BioAid. The BioAid algorithm is open source and available on GitHub [14]. In this manuscript, the replica of MATLAB code for BioAid is developed in Python. The BioAid algorithm was chosen above many other algorithms for smartphone based HAD apps because it is a complete computer model that reflects the biological functions of the human ear. The core model was made up of a series of equations that simulated the responses of the stapes, the basilar membrane (BM), outer and inner hair cells (OHCs, IHCs), the auditory nerve, and efferent activities such the acoustic reflex and the medial olivo-cochlear (MOC) system [15].

**Figure 1:**
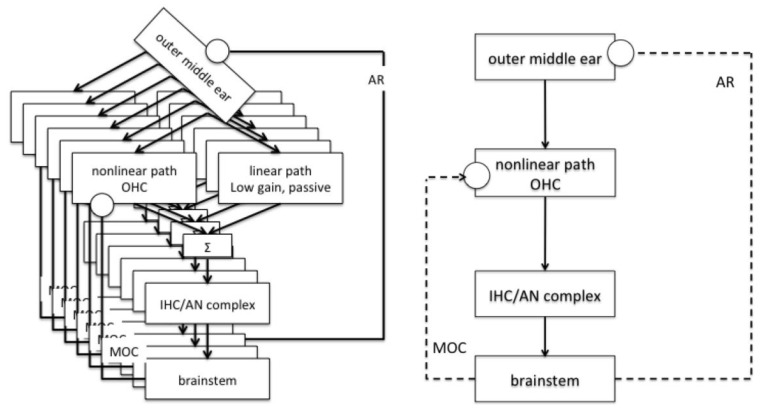
Flow diagram of the MATLAB Auditory Periphery (MAP) model taken from [15].

The BioAid algorithm was developed by a team of audiologists led by Professor Ray Meddis and computer scientists at the University of Essex, UK to produce a comprehensive computer model with numerous tuning options. The models developed were sophisticated and reliable enough to represent physical phenomena such as thresholds, masking, and speech recognition basics. The filter bank has six presets with 250 Hz to 8 kHz frequencies. AR, MOC, and Instantaneous compression regulate audio input and respond to loudness changes. The AR controls low-frequency sound and activates at a 70 dB SPL [15]. Instantaneous Compression attenuates unexpected sound level rises without the use of automated gain control (AGC) [15]. While the MOC acts as negative feedback, filtering out any noise caused by the compression process as shown in Figure 1. This application does not boost all audio levels with a specified frequency, which would make loud noises more uncomfortable. BioAid combines two physiologically inspired processing algorithms (normal and impaired hearing) from a computer model to build a HA. BioAid consists of a filter bank, an Acoustic Reflex (AR) system, a MOC feedback system, and a basilar membrane compression [16].

To the best of the authors’ knowledge, there is no intelligent system that can “scan” its surroundings and auto-adjust the HAD based on user preferences against a specific auditory scenario. The goal of this research is to design a smart HAD based on dedicated signal processing and machine learning. This research is an improvement on an already-in-use HAD known as the BioAid (newer version AUD1) for patients with mild to moderate hearing loss. The goal of this research is to enhance the capabilities of BioAid so that it automatically determines the user’s preference based on the current auditory scene that the user is in at the moment. The major goal of this research is to automate the smart HA with scene categorization so that the system can learn the user’s preferences for a particular setting. The system was trained by monitoring user preferences in relation to each scenario.

## 2. Literature Review

This section addresses some of the great advances in the HA industry, as well as approaches for acoustic scene classification.

### 2.1. Hearing Assistive Devices

HADs help to compensate for the impairments caused by hearing loss. There are numerous weaknesses to overcome with sensorineural hearing loss. Some sounds are completely inaudible while other sounds can be heard because a portion of their spectra is still audible, but may not be accurately identified since portions of their spectra (often the high-frequency portions) are inaudible [17]. A person with sensorineural hearing loss has a narrower range of levels between the weakest sound that can be heard and the most intense sound that can be endured than a person with normal hearing [17]. In order to compensate for this, hearing aids and hearing assistive devices (HADs) must enhance weak sounds over loud noises.

Furthermore, sensorineural dysfunction impairs a person’s capacity to identify and evaluate energy at one frequency in the presence of energy at other frequencies [17]. To overcome these issues, different investigators have proposed HAs and HADs.

A smart HAD that provides much-needed aid with speech interpretation in noisy surroundings was presented by [18]. The user must set the microphone array-based equipment in front of him/her on a flat surface, such as a table. When conversations begin, the microphone array analyzes sound from all directions, prioritizing the primary speaker’s voice while suppressing undesired noises. Using speech algorithms, this device automatically selects and switches between speakers. The user can fine-tune acoustic parameters using a smartphone interface.

In [19] the authors developed a smart earplug framework combined with non-intrusive bone conduction technology capable of enhancing speech with audio processing and filtering noise from audio. The design mechanism of these HADs simply absorbs sound energy and directs it to the ear canal at increased levels. Under the suggested method, individuals with conductive or mixed hearing loss who are unable to use air conduction (AC) HADs may use bone conduction devices (BCDs). Additionally, the system acts as an integrated music player, a live activity tracker, and an alert system for incoming messages on the user’s phone. In terms of both concrete applications and simulations in real time, the system performs well.

The structure of HAD proposed by [20,21] comprises primarily three components: earpieces, mobile computing platform, and real-time speech-enhancement application. Complex algorithms can be executed without straining the HAD’s processors. The complex and power-consumptive binaural algorithm is used to improve user experience. The speech-enhancement method was simplified compared to typical HADs with integrated digital signal processing. The 400-MHz transceiver helps reduce route loss around the body when combined with a HAD and mobile computing platform. Signal-to-noise ratios have improved by at least 30% in some conditions, and the total system delay was 8.8 milliseconds. Objective and subjective results show that the recommended structure, presented in [20] improves user experience.

In [21], the microphone of the earpiece was used to acquire the speaker’s voice, which was then wirelessly sent to the smartphone. After the deep learning speech enhancement application improves the speech intelligibility in real time, it then returned to the earpiece to make sound. The results demonstrated that the average utilization of the smartphone’s central processing unit was roughly 26%, and the signal-to-noise ratios improved by at least 20%. The provided empirical and subjective results demonstrate that the proposed strategy [21] produces superior noise suppression without audio distortion.

A smartphone-based context recognition system that uses sensory data to determine a scenario and modify hearing aid parameters was presented by [22]. The model’s primary components are two context reasoning methods (scene recognition and activity recognition). For scene recognition, according to the Google Nearby (GN) Searching API, 500 location points from nine classes were collected in Shenzhen. Each record in the dataset contains the user’s longitude, latitude, manually marked type, and the location points of all nearby places retrieved by the GN location searching interface. Neural network (NN), KNN, SVM, C4.5, and Naïve Bayes are trained and evaluated with C4.5 achieving 88.89 % accuracy. A mobile device with integrated sensors was used to determine the mobility state of users. In the experiment, both acceleration and direction sensors are used to collect data from four classes namely, walking, running, still, and riding. Decision trees (C4.5, CHAID, and CART), SVM, and NN are trained and tested with CHAID achieving an accuracy of 73.84% in activity recognition. After identifying the scene and activities, the smartphone sends a Bluetooth signal to the hearing aid to initiate the amplification process.

The investigation in [23] explores the classification of “music,” “noise,” “speech with noise,” “silence,” and “clean speech” that can be used for automatic transition between different HAD algorithms depending on an auditory-related scene. The datasets are extracted from multiple databases for use in training and testing. One such database is free sound, which maintains more than 40 thousand sounds of different classes. The Noisy Speech Database is a second database that contains both clean and noisy speech. Artificial noise is intentionally added to clean speech to create speech with noise class for the CSTR VCTK Corpus database. The music data are obtained from the publicly accessible Million Song Database, which contains millions of recordings with audio features and metadata. The LibriSpeech ASR corpus is a database containing one thousand hours of 16 kHz clean English speech read aloud. The dataset is constructed from suitable English-read audiobooks. The silent dataset is obtained from YouTube by downloading video/audio, importing it into MATLAB, converting it to wav file, then clipping it for 5 s. Mel frequency cepstral coefficient (MFCC), Mel-spectrogram, Chroma, Spectral contrast, and Tonnetz were retrieved from the audio of the following five classes: “music,” “noise,” “speech with noise,” “silence,” and “clean speech”. The convolution neural network (CNN) processed audio using these features. The system has an accuracy of 93.84%. The algorithm is efficient and uses a small amount of memory.

A smart HAD employing a deep neural network is used to enhance three specific sounds namely a fire alarm, a car horn, and a baby cry is presented in [24]. This is a significant contribution to people suffering from hearing loss to avoid catastrophic occurrences. AURIS, a portable smart space interface, was developed by the Cochlear Implant Processing Lab (CILab) at UT-Dallas [25]. The proposed AURIS interface samples the auditory space on a regular basis and uses a learn-versus-test phase to develop a Gaussian mixture model for each given ambient location. The AURIS interface connects to the CRSS CCi-Mobile research platform via an Android app to fine-tune the configuration parameters for cochlear implant (CI) or HAD users entering the room/location. Baseline objective evaluations were performed using 12 h of audio recordings in a range of naturalistic scenarios. At the University of Texas at Dallas, audio was collected at Starbucks, the library, Synergy Park North (SPN-UTDesign Studio), Chick-Fil-A, the pool tables area in the Student Union, the Solarium Lab, the gym, the Electrical and Computer Science-North (ECSN) study area, and the Electrical and Computer Science-South (ECSS) classroom. The performance metrics are determined by verified wireless communication, as well as estimated acoustic environment knowledge and more than 90% accuracy in room classification.

### 2.2. Scene Classification

A novel approach for acoustic scene classification (ASC) using a deep neural decision forest (DNDF) was proposed by [26]. DNDF combines convolutional layers and a decision forest as the final classifier. The experimental findings on the DCASE2019 and ESC-50 datasets show that the suggested DNDF approach increases ASC performance in terms of accuracy by 75.90% and 88.90%, respectively.

An ensemble classifier-based technique for ASC was presented by [27]. First, the signal was separated into the left and right mono channels, and then feature extraction was carried out by applying wavelet scattering individually to the left and right channels. Using these features, a separate random subspace classifier is trained for each mono (left and right) channel. Each classifier’s output was sent to the fusion stage, which combines them nonlinearly to improve accuracy. The parameters of this step were selected using a genetic algorithm. This [27] technique has a greater classification accuracy of 95% on the Dcase2017 dataset.

An acoustic spectrum transformation network that changed typical log-mel spectrums into imagined visual features (IVF) was presented by [28]. The anticipated visual elements were learned by taking advantage of the link between auditory and visual aspects in video recordings. Images were encoded as visual features using an auto-encoder, and a transformation network was learned to produce imagined visual features from log-mel. A big dataset of YouTube videos was used to train the model. The suggested technique was tested on the DCASE and ESC-50 scene classification tasks, and it outperforms existing spectrum characteristics with an accuracy of 83.7%.

A method for investigating the advantages of deep scalogram representations, extracted in segments from an audio stream was presented by [29]. The presented method first converts segmented acoustic scenes into bump and morse scalograms, as well as spectrograms; second, the spectrograms or scalograms are fed into pre-trained convolutional neural networks; third, the features extracted from a subsequent fully connected layer are fed into (bidirectional) gated recurrent neural networks, which are then followed by a single highway layer and a softmax layer; and finally perform predictions. The suggested technique is then evaluated using the acoustic scene classification dataset from the DCASE2017. When fusing the spectrogram and the bump scalogram on the evaluation set, bidirectional gated recurrent neural networks achieve an accuracy of 64%, an increase over the DCASE 2017 baseline result of 61%.

For acoustic scene classification (ASC), an alternative representation framework to the typically used time–frequency format was presented [30]. A pre-trained CNN with its different intermediary layers was used to represent a raw audio stream. The study assumes that the representations obtained from the intermediate layers are fundamentally low-dimensional. PCA was used to produce low-dimensional embeddings. The underlying subspace is approximated using an artificial dictionary learning approach. Furthermore, under the ensemble framework, the low-dimensional embeddings were aggregated in a late-fusion way to integrate hierarchical information gained at multiple intermediary levels. On a pre-trained 1-D CNN, SoundNet, the experimental evaluation was performed on publicly accessible DCASE 2017 and 2018 ASC datasets. Deeper layers, empirically, have a higher compression ratio than others. The performance was comparable to that obtained without applying any dimensionality reduction at a compression ratio of 70% across diverse datasets. The suggested framework outperforms techniques based on time–frequency representation.

From the literature, it is evident that multiple researchers presented HADs that enable hearing-impaired individuals. To deal with changes in environment and noise, the user has to manually adjust and tune the device. This is a tedious and time-consuming practice. Some of these HADs just amplify noises, including background noise, so the individual cannot hear clearly. Some researchers classified acoustic scenes with a maximum accuracy of 95% using the DCASE dataset. A system was needed to eliminate the manual switching of the parameters. therefore, [22,25] proposed a system that classifies scenes and adjusts the HAD settings appropriately. The [22] uses the user’s longitude and latitude parameters in conjunction with the Google API to determine the user’s location. The method proposed in [22] only amplifies the audio after identifying the scene/location. The research presented in [25] gathers audio data in several university classrooms, classifies it, and then automatically adjusts the HAD’s settings. The technique shown in [25] is limited to just a small number of interior scenes due to the need for an AURIS device that gathers the acoustic scene of the rooms. The study presented in this manuscript aims to eliminate this manual switching and automate the BioAid (a HAD) using a scene classification algorithm so that the system automatically recognizes the user-selected preferences based on adequate training. For scene classification a benchmark dataset DCASE 2017 was used and merged with the BioAid.

## 3. Methodology

The original BioAid algorithm was developed in MATLAB; the goal of the proposed approach for the automatic user preferences selection of smart HAD system adapts the BioAid algorithm code into Python.

The proposed system consists of two phases, the first comprises of scene classification model and the second phase is to fuse scene classifier with BioAid. In the first phase, a classifier is developed for the auditory scene classification and fused with the BioAid algorithm to automate presets and subsets selection for the BioAid based on the auditory scene. The TUT Acoustic Scenes 2017 dataset [31] from DCASE “Database for acoustic scene classification and sound event detection” was used for scene classification. A lakeside beach, a bus, a car, a café–restaurant, a city center, a forest trail, a grocery store, a home, a library, a metro station, a train, a tram, an office, an urban park, and a residential neighborhood are among the 15 audio scenes comprises acoustic dataset. Each scene has 312 audio files. These scenes depict a diverse range of real-world sites and are split into two broad environmental categories: “indoor” and “outdoor”.

Fifteen (15) audio scenes have 312 audio files of 10 s duration each. The audio files were in “.au” format, but in order to extract features, they were converted to “.wav” format before being imported into Python. To obtain MFCC features, the audio signals were segmented and windowed into 40 ms short frames. MFCC features with 12 coefficients were extracted using Librosa, a Python library, with pre-defined FFT and Mel-scale parameters. Subsequently, the particular acoustic scene was divided into 432 frames with a window length of 1024 and a hop size of 512. A 10 s acoustic audio signal has 432 frames. This results in matrix “C” with 432 rows and 12 columns for a single audio file. Here, the rows are frames, and 12 columns are 12 MFCC coefficients. The final feature vector space “D” of size 12 is obtained as follows:

(1) $$F=\left[\frac{1}{N}\overset{432}{\underset{i=1}{\sum }}a_{i1},\frac{1}{N}\overset{432}{\underset{i=1}{\sum }}a_{i2},\frac{1}{N}\overset{432}{\underset{i=1}{\sum }}a_{i3},\ldots \ldots \ldots \frac{1}{N}\overset{432}{\underset{i=1}{\sum }}a_{i12}\right]$$

where *i* is the *i*th frame and *N* is the total number of frames, i.e., 432. The 13 entries of dataset are 12 MFCC coefficients and the last entry is the label of that scene. Subsequently, the D for all audio scenes is calculated and forms a matrix A, the final dataset.

Matrix A has the following size:

Number of rows = 15 × 312 = 4680

where 15 is the total number of scenes and 312 is the total number of files in each scene.

Number of columns = 13

where 12 is MFCC coefficients and the last entry is for scene label.

The same procedure is applied with 40 MFCC features to obtain another dataset B.

**Figure 2:**
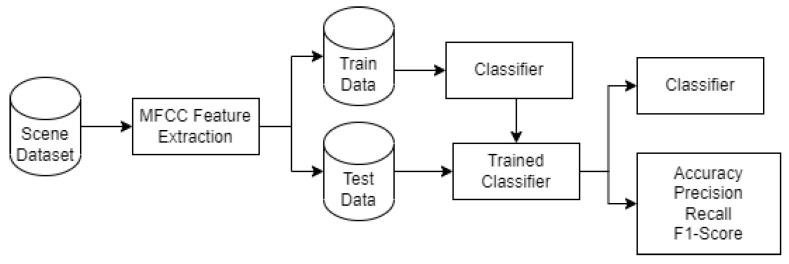
Scene classification model.

**Figure 3:**
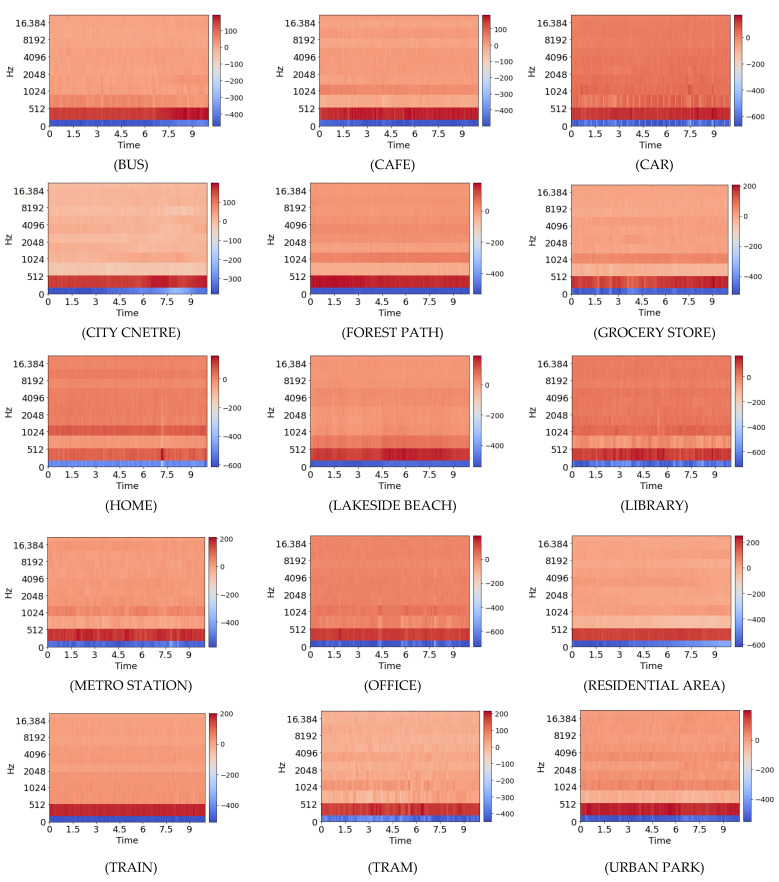
Plot of Mel frequency cepstral coefficient (MFCC).

Figure 2 shows the scene classification model pipeline. Figure 3 shows the plots of MFCC coefficients of all scenes from the dataset to demonstrate how effective MFCCs are at extracting audio information. Dataset A was then separated into a training set and a test set. On this dataset, various machine learning (ML) models that include random forest (RF), multi-layer perceptron (MLP), extra tree classifier (ETC), and K-nearest neighbor (KNN) were trained and tested on the datasets. The RF classifier that achieved highest level of generalization with an accuracy of 99.7% was selected.

**Figure 4:**
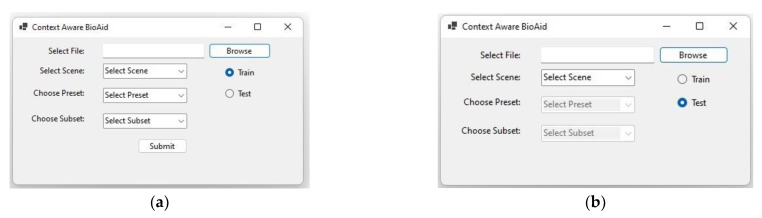
GUI of context aware BioAid system (**a**) training phase (**b**) testing phase.

**Figure 5:**
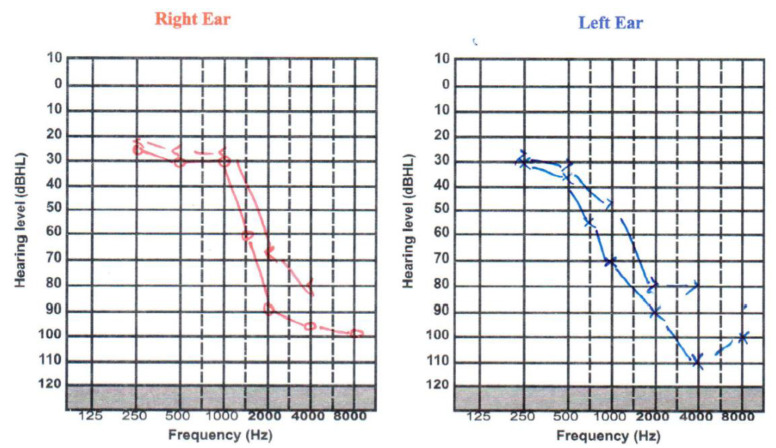
Subject audiogram chart.

Multiple clean speeches taken from LJ speech dataset were convolved with scenes from the DCASE dataset. LJ speech dataset is a collection of 13,100 short audio clips of a single speaker reading lines from seven non-fiction books. In the 2nd phase, the scene classifier is fused with BioAid algorithm such that the predicted scene from scene classifier is fed into BioAid. The whole system works in two stages, training and testing. A GUI is designed as shown in Figure 4 to select any stage. A participant is asked to listen to the convolved audios and select the best tuning combination of presets and subsets parameters for BioAid based on participant experience. Ethical approval for this work was sought and approved by the Khwaja Fareed University of Engineering and Information Technology. The hearing impairment in a 31-year-old male patient was examined using standard audiometry and tympanogram tests with the range of frequency and level in hospital under the supervision of an audiologist and obtained the audiogram as shown in Figure 5.

A pure-tone audiometry test measures the softest, or least audible, sound that a person can hear. The loudness of sound is measured in decibels (dB). A whisper is about 20 dB, loud music ranges from 80–120 dB, and a jet engine is about 180 dB, for reference. The tone of sound is measured in frequencies (Hz). Low bass tones range from 50–60 Hz, high-pitched tones range from 10,000 Hz or higher. A normal hearing range is 250–8000 Hz at 25 dB or lower. Figure 5 shows hearing loss in high frequency ranges from 1 KHz to 8 KHz.

The participant selection of presets and subset combinations against the scene is stored in CSV file. This file will then serve as training data for ultimate model for selection of combination of presets and subsets based on the scene. This task was performed multiple times with different convolved (speech and scene) audio input against each scene and subsequently recorded. The recording process is carried out for all 15 scenes.

**Figure 6:**
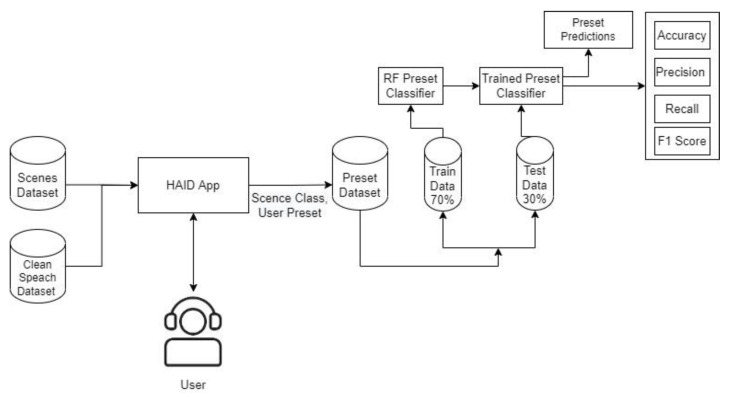
Pre-set training model.

A structured dataset (user preferences dataset) was maintained that contains the combination of presets and subsets as labels against each instance of scene. The combination of presets and subsets is based on user preferences against each scene. Different ML classifiers were trained and tested on the user preferences dataset as shown in Figure 6.

**Figure 7:**
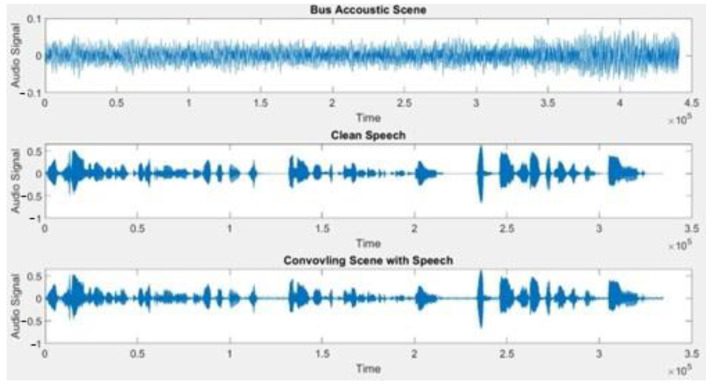
Scene, speech, and convolved signal of scene with speech.

**Figure 8:**
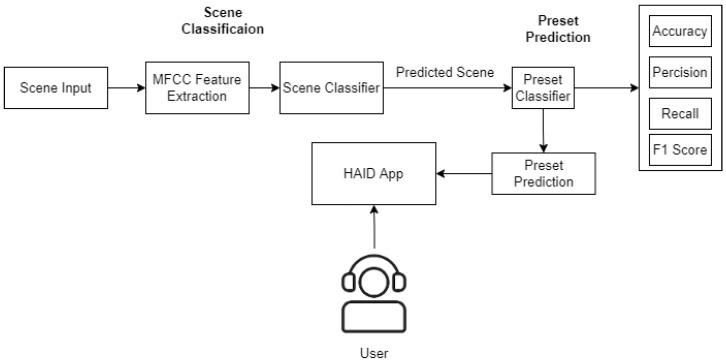
User preference selection.

Post-training, the testing stage is in place. In the testing phase, a clean speech file is convolved with the audio of a random scene, and the presets and subsets are automatically configured in BioAid. Since the pauses in speech signal correspond to acoustic scene, the MFCC features are extracted from the pauses presented in the input signal and are sent through a pretrained scene classifier to forecast the scene in the input audio. Figure 7 shows the speech and scene convolved signal. Subsequently, the predicted scene is sent into another classifier that predicts the combination of presets and subsets based on that particular user’s previous history of preset and subset choices vs the specific scene. The predicted preset and subset are automatically tuned in the BioAid. The steps involved in the automatic preset and subset selection are shown in Figure 8.

## 4. Results

This section focuses on the results and performance of the different classifiers.

### 4.1. Results of Acoustic Scene Classification

The 12 and 40 MFCC features were retrieved from the audio dataset and labeled datasets were maintained. The datasets were divided into 70% training data and 30% testing data. Hyperparameters used to tune the classifiers are given in Table 1. Using a grid search strategy, the function output for each of these hyperparameter values was calculated and the most effective hyperparameters in terms of best accuracy for the available dataset were chosen.

**Figure 9:**
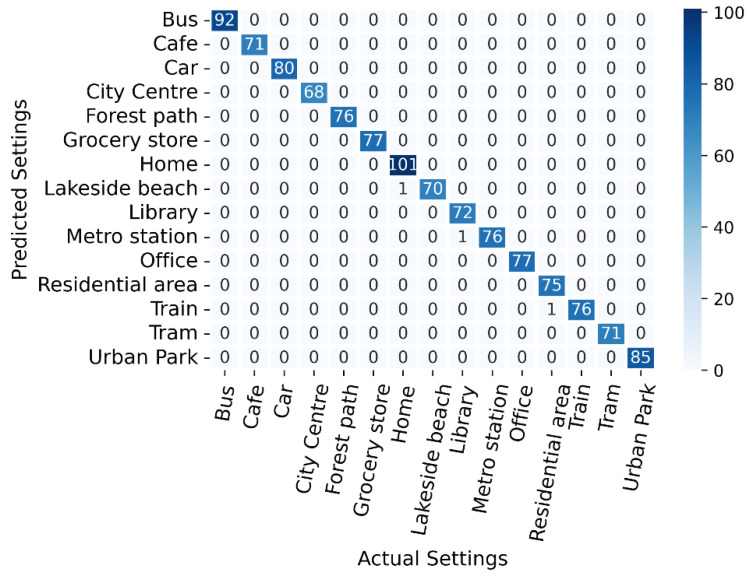
Confusion matrix of scene classification with 12 MFCC features.

The dataset containing 12 MFCC features yields better results and is shown in Table 2. RF performs better on this dataset than other ML models because tree-based classifiers perform better on multiclass datasets [32,33]. RF trained with 12 MFCC features was selected for scene classification. Confusion matrix of RF with 12 MFCC features can be seen in Figure 9. The confusion matrix reveals that RF incorrectly predicted the lakeside beach, train station, and metro station once.

### 4.2. Results of Preset Selection 

**Figure 10:**
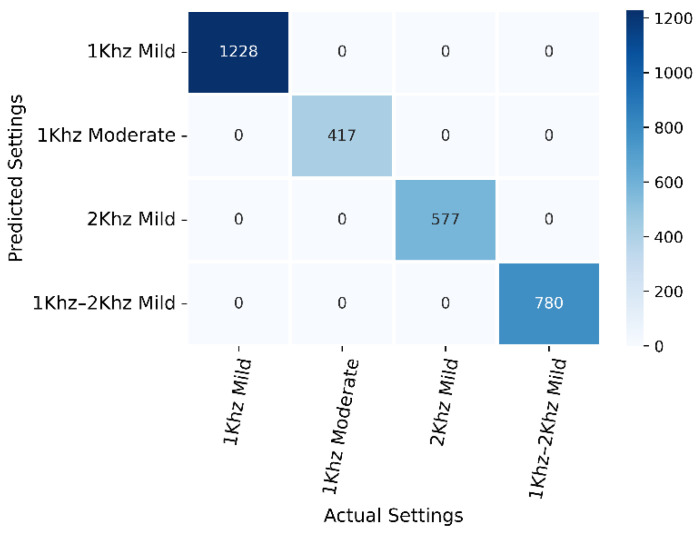
Confusion matrix of preset and subset selection.

The user’s preference dataset comprises 6006 rows and 2 columns, where columns represent the scene and user response of preset and subset, respectively. The dataset was then divided into a 70–30 ratio for training and testing. Several ML models were trained and evaluated but RF with tuning parameters (random_state = 142, max_depth = 50, n_estimators = 50) achieved an accuracy of 100%. The classification report is presented in Table 3 and the confusion matrix can be seen in Figure 10. It can be seen in the confusion matrix that there are only three classes, which means the user always selected from three choices out of twenty-four presets and subsets. The proposed system is user-centric. Each user will have his/her own dataset of presets and subsets choices against the acoustic scene. For any other user, s/he will have his/her own dataset.

Multiple users’ preferences will not comprise a single dataset. Subject B can get benefit from the dataset and the model of A if the two subjects have equal hearing impairments and acoustic scene scenarios.

**Figure 11:**
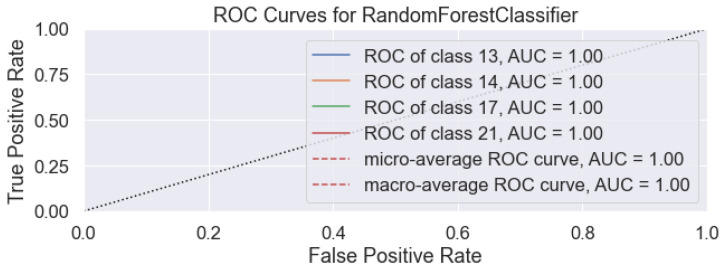
RF preset-subsets prediction performance evaluation using ROC curve.

The standard evaluation technique’s accuracy and ROC curve have been performed and are shown in Figure 11. Since the area under the curve (AUC) is 1.00 for all subject preference classes, the curves are at the top left corner and cannot be viewed since this coincides with the top left corner. The more the ROC curve is positioned towards the top left corner implies better model performance. The evaluation shows that the proposed system completely automates the preset and subset selection against scenes, as per subject preferences. The automated preset selection and subsets combination show 100% accuracy, based on the obtained datasets.

### 4.3. Comparison with Related Work

The proposed method is compared with techniques presented by different investigators. Although the system presented by [22] also performs scene classification and amplifies the parameters of HAD accordingly. The [22] relies on Google API for user localization using the user’s longitude and latitude parameters. The system presented by [22] only amplifies the signal once it has the scene/location identity. Work in [25] collects acoustic data in different rooms of a university setting and after classification autotunes the parameters of the HAD. The system presented by [25] needs an AURIS unit that collects the acoustic scene of the rooms and is thus limited to very few scenes mostly indoors. For the louder acoustic scene (>85dB), it only generates an alert while the BioAid has a built-in feature of instantaneous compression for an abrupt increase in the intensity in the acoustic signal. The datasets collected by [22,25] are small in size. In contrast, the proposed method employs a benchmark dataset containing audio from fifteen distinct scenes. It is evident from Table 4 that the proposed method outperformed in terms of accuracy in scene classification.

## 5. Conclusions

A hearing aid or hearing assistive device provides numerous benefits to those who need support with hearing loss, enabling them to freely converse and engage in daily living activities without fear or embarrassment. A context-aware BioAid prototype has been presented and is based on BioAid algorithm enhancement after its adaptation to Python. The proposed method comprises the classification of auditory scenes and the selection of BioAid tuning settings based on user experiences. An acoustic scene classification goal is to identify a specified acoustic signature that best reflects the recorded environment. Scene classification was performed using the DCASE2017 dataset. Multiple classifiers were trained and tested, with RF surpassing the others with an accuracy of 99.7%. In the second part, audio sets of clean speech from the LJ speech dataset were merged with scenes, and the user was asked to listen to the resulting audio sets and adjust their presets and subsets. A CSV file was maintained that contained the presets and subsets that the user could hear clearly. Various classifiers were trained using the user preferences dataset. Clean speech audio was convolved with the scene and given to the scene classifier that predicted the scene after training. The predicted scene was then sent to the preset classifier, which predicts the user’s preference for preset and subset. The BioAid is automatically tuned to the predicted choice. An accuracy of 100% was achieved by random forest in predicting presets and subsets.

## Figures and Tables

**Table 1:** Parameters used to tune classifiers.

Classifier	Hyperparameters
RF	Random_state = 0, max_depth = 150, n_estimators = 1000
MLP	hidden_layer_sizes = (350, 300, 200, 100), activation = “relu”, random_state = 0, max_iter = 500
ETC	n_estimators = 100, max_depth = 200, random_state = 0
KNN	Algorithm = “auto”, leaf_size = 5, metric = “minkowski”, metric_params = None, n_jobs = 1, n_neighbors = 3

**Table 2:** Classification results of classifiers with 12 MFCC features.

Classifier	Accuracy	Precision	Recall	F1-Score
RF	99.7%	1.00	1.00	1.00
MLP	96.2%	0.96	0.96	0.96
ETC	95.8%	0.96	0.95	0.95
KNN	89.5%	0.90	0.89	0.89

**Table 3:** Preset and subset selection accuracy of RF.

Precision	Recall	F1-Score
1.00	1.00	1.00
1.00	1.00	1.00
1.00	1.00	1.00

**Table 4:** Comparison of accuracies of different investigation.

Reference	Accuracy in Scene Classification
[22]	88.89%
[25]	90%
Proposed Method	99.7%

## Data Availability

Data will be presented on demand.
